# Regarding clinical and laboratory indicators predicting coagulase-negative staphylococci as a cause of bloodstream infection among children below five years of age admitted at a Tertiary Hospital in Dar es Salaam, Tanzania

**DOI:** 10.1080/07853890.2025.2543059

**Published:** 2025-08-05

**Authors:** Zainab Hafeez

**Affiliations:** Nishtar Medical University and Hospital, Multan, Pakistan

It was a pleasure to read a recent article on identifying clinical and laboratory indicators for diagnosing true coagulase-negative staphylococcal bloodstream infections (CoNS-BSI) in children under five [1]. CoNS often contaminates blood cultures due to its presence on the skin, which can lead to underdiagnosis when clinicians assume that all CoNS-positive blood cultures are contamination. On the other hand, overdiagnosis and unnecessary use of empiric antibiotics remain an issue in some settings, driving antimicrobial resistance, especially in low-resource settings where advanced diagnostics like multiplex PCR are not readily available.

This study highlights two clinical risk factors: tachycardia >170 bpm and *in situ* IV cannulation, alongside a key lab strategy: using two blood culture sets instead of one. When either risk factor is present, clinicians are advised to collect two sets to confirm true CoNS-BSI before starting antibiotics. This helps avoid overtreatment and limits the spread of resistance.

In short, this study offers a practical approach to diagnosing CoNS-BSI at the bedside. It addresses a highly relevant clinical challenge, and the authors deserve praise for their efforts to improve pediatric sepsis care in resource-limited settings. Future research could build on this by developing a validated clinical prediction score and testing its performance in a new cohort to enhance clinical decision-making.

One thing I would like to add, is the significance of assessing clinical outcomes from CoNS-BSI. Knowing whether CoNS-BSI leads to worse outcomes (such as mortality, ICU admission, and longer hospital stay) would help to further emphasize the clinical significance of developing a predictive scoring tool. A study by Julia Johnson et al. [2] reported a high rate of bloodstream infections (BSIs) linked to antimicrobial resistance and mortality in a NICU in Pune, India. Most infections were due to gram-negative bacteria (GNB), highlighting the urgent need for targeted measures to lower BSI rates and related deaths in hospitalized neonates. A similar inclusion of tracking of outcomes to show the extent of adverse events from CoNS-BSI, and its effect on prolonging recovery could justify efforts for early detection.

Another issue that needs to be addressed is a detailed analysis of the duration and site of IV cannulation. Not all IV cannulas carry equal risk. For example, the duration of use (number of days) and site of insertion (femoral, subclavian, internal jugular, or peripheral veins) are important. The collection of data on how long the IV cannula was in place, its location and handling practices could help tailor more precise catheter-related infection control measures.

Finally, I would like to add that more data is needed on infants under one month of age, especially premature and low birth weight babies. This should include the risk of CoNS-BSI with central versus peripheral catheters. Ainsworth et al. [3] found that central lines offer better nutrition delivery and reduce the need for multiple lines in neonates. These findings highlight the importance of comparing catheter types to guide safer, more effective line placement in neonates. Further research could clarify the risks and benefits of central versus peripheral catheters across different pediatric age groups.

## Data Availability

Data sharing is not applicable to this article as no data were created or analysed in this study.
